# Novel Epoxy Activated Hydrogels for Solving Lactose Intolerance

**DOI:** 10.1155/2014/817985

**Published:** 2014-06-11

**Authors:** Magdy M. M. Elnashar, Mohamed E. Hassan

**Affiliations:** ^1^Center of Excellence, Encapsulation & Nanobiotechnology Group, National Research Center, El-Behouth Street, Cairo 12311, Egypt; ^2^Polymers Department, National Research Center, El-Behouth Street, Cairo 12311, Egypt; ^3^Biochemistry Department, Taif University, Taif, Saudi Arabia; ^4^Chemistry of Natural and Microbial Products Department, National Research Center, El-Behouth Street, Cairo 12311, Egypt

## Abstract

“Lactose intolerance” is a medical problem for almost 70% of the world population. Milk and dairy products contain 5–10% w/v lactose. Hydrolysis of lactose by immobilized lactase is an industrial solution. In this work, we succeeded to increase the lactase loading capacity to more than 3-fold to 36.3 U/g gel using epoxy activated hydrogels compared to 11 U/g gel using aldehyde activated carrageenan. The hydrogel's mode of interaction was proven by FTIR, DSC, and TGA. The high activity of the epoxy group was regarded to its ability to attach to the enzyme's –SH, –NH, and –OH groups, whereas the aldehyde group could only bind to the enzyme's –NH_2_ group. The optimum conditions for immobilization such as epoxy chain length and enzyme concentration have been studied. Furthermore, the optimum enzyme conditions were also deliberated and showed better stability for the immobilized enzyme and the Michaelis constants, *K*_*m*_ and *V*_max_, were doubled. Results revealed also that both free and immobilized enzymes reached their maximum rate of lactose conversion after 2 h, albeit, the aldehyde activated hydrogel could only reach 63% of the free enzyme. In brief, the epoxy activated hydrogels are more efficient in immobilizing more enzymes than the aldehyde activated hydrogel.

## 1. Introduction

Lactases (*β*-galactosidases) are indeed important enzymes in food industry and have found significant applications in enhancing sweetness, solubility, flavor, and digestibility of dairy products [1]. A major application of *β*-galactosidase is lactose hydrolysis, a process that results in the formation of glucose and galactose. Lactose is the major sugar (4-5%) present in milk. The consumption of foods with a high content of lactose is causing a medical problem for almost 70% of the world population, especially in the developing countries, as the naturally present enzyme in the human intestine loses its activity during lifetime, so its hydrolysis makes milk fit for consumption of lactose intolerant people [2]. Hydrolysis of lactose present in milk using enzymes such as lactases will produce lactose-free milk and lactose-free dairy products [3].

In industries, immobilized enzymes are preferred over the free ones. The immobilization technique would enable the reusability of enzymes for tens of times, reducing the enzyme and product cost significantly. Unfortunately, efficient commercial carriers suitable for immobilization of enzymes are relatively expensive [4, 5]. Understandably, for food, pharmaceutical, medical, and agricultural applications, nontoxicity, and biocompatibility of the materials are also required. Among many carriers that have been considered and studied for immobilizing enzymes, organic or inorganic, natural or synthetic, chitosan and carrageenan are of interest in that they offer most of the above characteristics and are available at a reasonable cost [3].

Chitosan is a cationic naturally occurring polymer obtained from the deacetylation of chitin, which is the second most abundant polymer in nature after cellulose [6, 7]. Chitosan has an abundance of amino groups (70–95%), that make chitosan a cationic polyelectrolyte (pKa ≈ 6.5) and one of the few found in nature. This basicity gives chitosan singular properties: chitosan is soluble in aqueous acidic media at pH < 6.5 and when dissolved possesses high positive charge on NH_3_^+^ groups. The protonated (NH_3_^+^) could adhere to negatively charged surfaces such as polyanionic compounds [8, 9].

Carrageenan is a naturally occurring anionic polysaccharide isolated from the seaweeds. According to the study made by Chao et al., 1986 [10], to harden the carrageenan gels using different amine compounds, they concluded that only polyamines such as chitosan substantially improved the carrageenan gels thermal stability via polyelectrolyte's interactions. Unfortunately, the carrageenan-polyelectrolyte systems were limited to the entrapment of enzymes [11–13], which have the major problem of enzyme leakage. For example, Boadi and Neufeld, 2001 [14], used alginate and carrageenan to entrap tannase and then crosslinked the gel beads with chitosan followed by glutaraldehyde. The entrapment technique limits their industrial use as supports for enzyme immobilization due to enzyme leakage. So, efforts to immobilize enzymes on newer type of carriers, especially with covalent bonds, are still underway in many laboratories [15–18].

To our knowledge, previous reports have not any greater extent that dealt with carrageenan-chitosan polyelectrolyte as a use for the covalent immobilization of enzymes, with an exception of our recent work [19, 20]. In that work, we immobilized *β*-galactosidase covalently via its amino groups to carrageenan treated with chitosan and glutaraldehyde. Although the linkage was covalent, however, the enzyme loading capacity was limited to up to 11 U/g gel beads. This could be regarded to the free aldehyde groups on the carrageenan treated chitosan that could only react with the free amino groups (NH_2_) on the enzyme via Schiff's base formation, –C=NH– [19, 20].

Thus, the purpose of this research was to modify the carrageenan with chitosan and a more efficient functional group, epoxy group, which imparts three extra benefits to carrageenan:

- the first is improvement of the carrageenan gel's thermal stability by forming a polyelectrolyte complex (PEC) between the carrageenan –OSO_3_^−^ and the chitosan –NH_3_^+^;
- the second is creation of a new functionality, free chitosan amino groups (NH_2_);
- the third is activation of the free chitosan amino groups (–NH_2_) with 1,4-butanediol diglycidyl ether to impart free epoxy groups to covalently immobilize *β*-galactosidase via the –SH, –OH, and –NH of their amino acids.

Accordingly, we expect more enzymes to bind to the epoxy activated carrier via three groups on the enzyme (–SH, –OH, and –NH), whereas the free aldehyde groups could only bind to the –NH_2_ of the amino acids. To our knowledge, there are no reports on the use of carrageenan for the immobilization of *β*-galactosidase using covalent technique via chitosan and 1,4-butanediol diglycidyl ether. The novel gel formulation was prepared in beads shape using the Encapsulator to enable gel beads production on the semipilot scale and to increase the gel's surface area. The grafted formulation was illustrated using a schematic diagram and the chemical and thermal modification was proved using the FTIR and DSC techniques, respectively. On the other hand, the enzyme loading capacity was optimized by using short and long chain of the epoxy activated carrier and using different concentrations of the enzyme. Finally, the free and immobilized enzymes were characterized for their activities at different pHs and temperatures and the Michaelis constants were studied as well as the lactose hydrolysis using free and immobilized enzyme over time.

## 2. Materials and Methods

As a general rule, all experiments were carried out in triplicate and data are means ± SD (*n* = 3). The abbreviations section is listing all the abbreviations used in the formulations samples.

### 2.1. Materials

*κ*-Carrageenan (MW: 154,000), sulfate ester ~25, and chitosan were supplied by Fluka. *β*-Galactosidase (EC 3.2.1.23) was obtained from Aspergillus oryzae and 11.8 U/mg was obtained from Sigma-Fluka-Aldrich. All other chemicals were of pure grades (Analar or equivalent quality). The Encapsulator, model IE-50, was purchased from Innotech Encapsulator in Switzerland. The gel disks dimensions were measured using a micrometer (Micro 2000, 0–25 mm).

### 2.2. Determination of *β*-Galactosidase Activity

*β*-Galactosidase activity was determined by the rate of glucose formation in the reaction medium. Known amount of immobilized or free enzyme was incubated into 10 mL of 200 mM lactose solution in 100 mM citrate phosphate buffer at pH 4.5 for 3 h at 37°C and 100 rpm. At the end of the time, 50 *μ*L of reaction mixture was added to 950 *μ*L buffer and boiled for 10 min to inactivate the enzyme and analyzed for glucose content using the glucose test. One enzyme unit (IU) was defined as the amount of enzyme that catalyzes the formation of 1 *μ*mol of glucose per minute under the specified conditions.

Equations (1), (2), and (3) show the hydrolysis of lactose by *β*-galactosidase and glucose determination using a mixture of enzymes, glucose oxidase (GOD), and peroxidase (POD):

(1) $$\begin{array}{c} \text{Lactose}\overset{\beta \text{-galactosidase}}{\to }\text{Glucose}+\text{Galactose} \end{array}$$

(2) $$\begin{array}{c} \text{Glucose}+{\text{O}}_{2}+{\text{H}}_{2}\text{O}\overset{\text{GOD}}{\to }\text{Glucolactone}+{\text{H}}_{2}{\text{O}}_{2} \end{array}$$

(3) H2O2+Hydroxybenoate-Na-4-aminoantipyrine →PODQuinon  complex+H2O

Glucose concentration was measured spectrophotometrically with a glucose test based on the Trinder reagent. Glucose is transformed to gluconic acid and hydrogen peroxide by glucose oxidase (GOD). The hydrogen peroxide formed reacts in the presence of peroxidase (POD) with 4-aminoantipyrine and p-hydroxybenzene sulfonate to form a quinoneimine dye, as shown in Equations (1), (2), and (3).

The intensity of the color produced is directly proportional to the glucose concentration in the sample. The assay was performed by mixing 30 *μ*L of a sample of unknown concentration and 3 mL of Trinder reagent; the reaction was allowed to proceed for 20 min at room temperature, and the absorbance of the unknown concentration was read at 510 nm [19].

### 2.3. Preparation of Gel Beads

*κ*-Carrageenan gel was prepared by dissolving 2.5% (w/v) carrageenan in distilled water at 70°C using an overhead mechanical stirrer until complete dissolution had occurred. The Carrageenan gel solution was dropped through a nozzle of 300 *μ*m using the Innotech Encapsulator in a hardening solution of 0.3 M KCl. Then, beads were hardened using 0.3 M KCl for 3 h.

### 2.4. Modification of Gel Beads Using Chitosan and Epoxy

Two activated epoxy hydrogels were prepared, the short and the long chain, as follows.

- *The Short Chain*. Carr.-Ch.-Epo: beads were soaked in a solution of 0.75% chitosan previously prepared in 1% (v/v) acetic acid. Then, they were suspended in 75 mL 0.5 M NaOH containing 150 mg sodium cyanoborohydride under stirring. Slowly 75 mL 1, 4-butanediol diglycidyl ether was added with constant stirring and the reaction was left at room temperature overnight. Finally, the activated gel beads were extensively washed with water to remove excess reagent.
- *The Long Chain*. Carr.-Ch.-Epo.-Ch.-Epo: formula (a) was further modified with chitosan and then epoxy as shown above.

### 2.5. Elucidation of the Modified Gel Using Fourier Transform Infrared Spectroscopy

The infrared spectra of all formulations were recorded with Fourier transform infrared spectroscopy (FTIR-8300, Shimadzu, Japan). FTIR spectra were taken in the wavelength region 4,000 to 400 cm^−1^ at ambient temperature. The FTIR spectrophotometer (FTIR-8300, Shimadzu, Japan) was used to prove the presence of the new functional group, epoxy groups, in both forms of the modified gels. Five samples were used for this test: carrageenan (Carr.); carrageenan coated with chitosan (Carr.-Ch.); carrageenan coated with chitosan followed by epoxy (short chain epoxy activated hydrogel: Carr.-Ch.-Epo.); carrageenan coated with chitosan followed by epoxy followed by chitosan (Carr.-Ch-Epo.-Ch.); and, finally, carrageenan coated with chitosan followed by epoxy followed by chitosan followed by epoxy (long chain epoxy activated hydrogel: Carr.-Ch-Epo.-Ch.-Epo.).

A total of 2% (w/w) of the sample, with respect to the potassium bromide (KBr; S. D. Fine Chem, Ltd.) disk, was mixed with dry KBr. The mixture was ground into a fine powder using an agate mortar before it was compressed into a KBr disk under a hydraulic press at 10,000 psi. Each KBr disk was scanned 16 times at 4 mm/s at a resolution of 2 cm^−1^ over a wavenumber range of 400–4000 cm^−1^, using Happ-Genzel apodization.

### 2.6. Differential Scanning Calorimetry and Thermal Gravimetric Analysis

Differential scanning calorimetry (DSC) and thermal gravimetric analysis (TGAA) were performed to prove the formation of a strong polyelectrolyte complex between carrageenan and chitosan followed by di-epoxy. The thermal behavior of five gel formulations was performed: Carr.; Carr.-Ch; Carr.-Ch-Epo.; Carr.-Ch-Epo.-Ch.; Carr.-Ch-Epo.-Ch.-Epo. The differential scanning calorimetry was studied using DSC (SDT 600, TA Instruments, USA). Approximately 3 to 6 mg of the dried gels was weighed into an alumina pan. The samples were heated from 40°C to 340°C at a heating rate of 10°C/min. The thermal behavior of the different gel formulations was characterized for their TGA (SDT 600, TA Instruments, USA). Alumina pans were used and approximately 3 to 6 mg of the dried gels were weighed. The samples were heated from 50 to 300°C at a heating rate of 10°C/min.

### 2.7. Immobilization of *β*-Galactosidase

*β*-Galactosidase was immobilized onto the short and long chain of the epoxy activated hydrogels as follows. One gram of the activated gel beads was washed thoroughly with distilled water and was incubated into 10 mL of enzyme solution (3.0 U/mL) prepared in 100 mM citrate-phosphate buffer at pH 4.5 for 16 h. The immobilized enzyme was washed thoroughly with the buffer solution containing Tris-HCl to block any free aldehyde group and to remove any unbound enzyme. The immobilized enzyme was stored at 4°C for further measurements. Two parameters were used to reach the enzyme's maximum loading capacity (E.L.C.) and epoxy activated long and short chains as well as the enzyme concentration.

The E.L.C. or the amount of enzymes' units immobilized onto and into gel beads was calculated as follows:

(4) $$\begin{array}{c} \text{E}.\text{L}.\text{C}.\;=\frac{\left(M_{o}-M_{f}\right)}{W}, \end{array}$$

where *M*_*o*_ is the initial enzyme activity (U), *M*_*f*_ is the enzyme activity of the filtrate (U) after immobilization, and *W* is the weight of gel beads (g).

### 2.8. Optimization and Evaluation of the Free and Immobilized *β*-Galactosidase

#### 2.8.1. Temperature and pH Profiles for the Free and Immobilized *β*-Galactosidase

The free and immobilized *β*-galactosidases were incubated into 10 mL of 200 mM lactose at temperatures from 30°C to 70°C for 3 hrs. The enzyme activity has been determined according to Section 2.2. The optimum temperature has been chosen to study the effect of pH where the free and immobilized *β*-galactosidases were incubated into 10 mL of 200 mM lactose at pH 3.0–9.0 at 37°C for 3 hrs.

#### 2.8.2. *K*_*m*_ and *V*_max⁡_ of the Free and Immobilized *β*-Galactosidase

The Michaelis-Menten kinetic models adequate for the description of the hydrolysis of lactose by the free and the immobilized enzyme; apparent *K*_*m*_ and *V*_max⁡_ of free and immobilized *β*-galactosidase were determined for lactose using the Hanes-Woolf plot method. Free and immobilized *β*-galactosidases were incubated into 10 mL of 25 to 200 mM at 37°C and pH 4.5 for 3 hrs under standard assay conditions.

#### 2.8.3. Lactose Hydrolysis Using Immobilized *β*-Galactosidase

To evaluate the efficiency of the immobilized enzyme, it was used for lactose hydrolysis using the optimum conditions obtained from above optimization for the free and immobilized enzymes.

## 3. Results and Discussion

### 3.1. Grafted Alginate Elucidation Structure

**Scheme 1:**
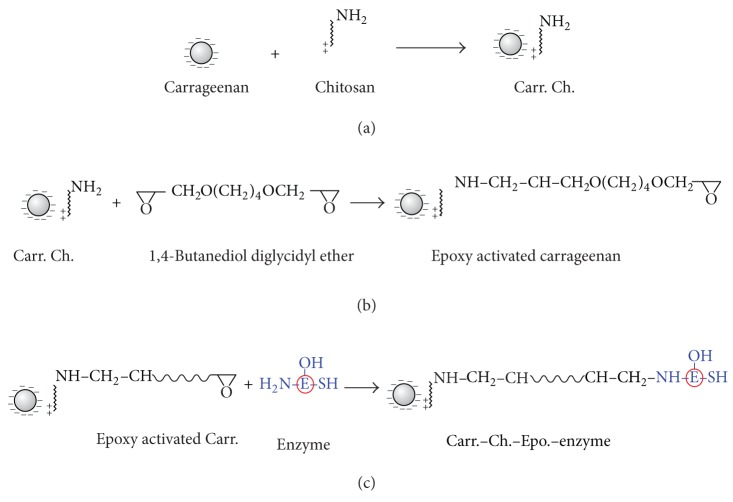
Grafted carrageenan gel beads with epoxy groups and immobilization of enzymes. (a) Modification of carrageenan beads with chitosan via ionic interaction. (b) Incorporation of epoxy groups to the carrageenan-chitosan. (c) Immobilization of enzymes to the grafted carrageenan-epoxy via –SH, –OH, or –NH_2_.

Protonated amino groups (–NH_3_^+^) of Ch formed a polyelectrolyte complex with the –OSO_3_^−^ of the Carr gel and incorporated free amino groups to Carr [19]. The free amino groups (–NH_2_) of Ch were used to covalently immobilize *β*-galactosidase via the di-epoxy groups as a mediator beside its main role as a cross-linker. The enzyme could be bound to the carrier's free epoxy groups via its –SH, –OH, and –NH groups. However, as shown in Scheme 1, we represented that one uses the free –NH groups as an example to follow the –OH and –SH groups.

**Figure 1:**
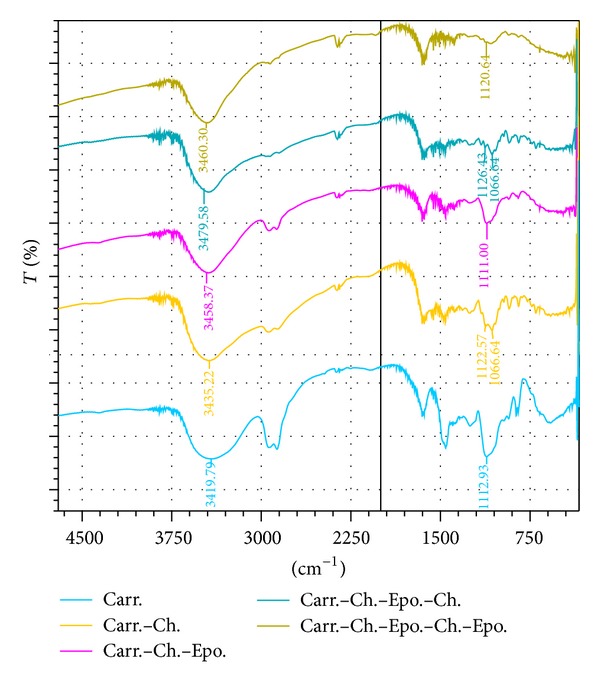
FTIR of five formulations of carrageenan and modified carrageenan with chitosan and epoxy groups: Carr; Carr-Ch; Carr-Ch-Epo; Carr-Ch-Epo-Ch; Carr-Ch-Epo-Ch-Epo.

The FTIR bands of Carr; Carr-Ch; Carr-Ch-Epo.; Carr-Ch-Epo-Ch; Carr-Ch-Epo-Ch-Epo were shown in Figure 1. Spectrums of the three compounds, Carr; Carr-Ch; Carr-Ch-Epo, revealed a new and strong band at 870 cm^−1^, which appears only for the modified gel spectrum with epoxy groups. This band proved the presence of a new gel functional group, epoxy group, which is in agreement with the author's previous work [20]. This band disappeared after treatment of the Carr-Ch-Epo with Ch and reappeared after further treatment with epoxy (Carr-Ch-Epo-Ch-Epo) at 880 cm^−1^. The FTIR bands also revealed a decrease in intensity and a shift of the –OSO_3_^−^ absorption band of Carr from 1446 cm^−1^ to 1390 cm^−1^after reaction with the chitosan. This ionic interaction between the carrageenan and the chitosan evidenced the formation of strong polyelectrolyte complexes [21].

**Figure 2:**
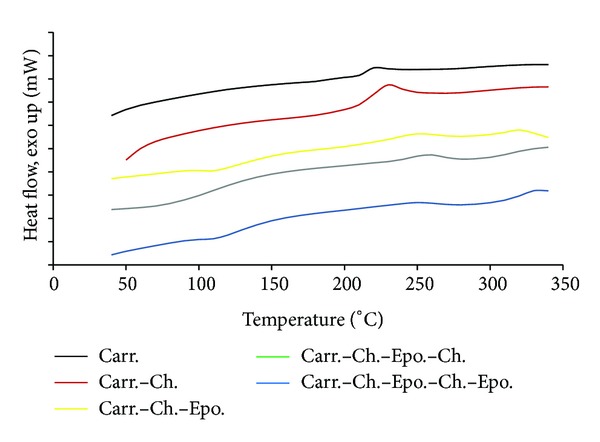
DSC thermogram of carrageenan and modified carrageenan with chitosan and epoxy groups.

**Figure 3:**
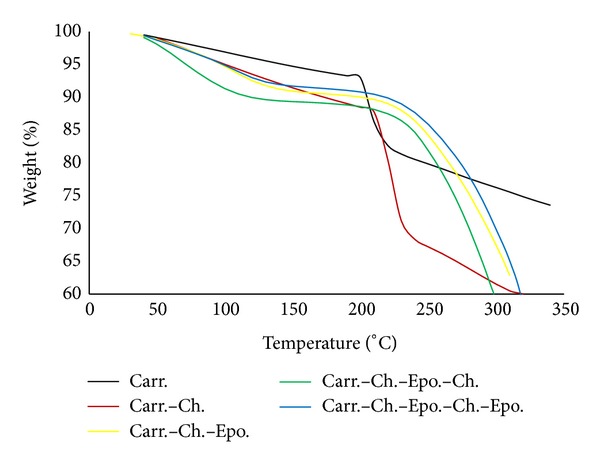
TGA thermogram of carrageenan and modified carrageenan with chitosan and epoxy groups.

Table 1 is tabulating the main characteristic values of the DSC and TGAA thermograms as shown in Figures 2 and 3, respectively.

The treatment of carrageenan with chitosan and epoxy has shown gradual and obvious improvement in their DSC and TGA. The DSC exothermic effect has been shifted to higher temperatures from formula numbers 1–5, that is, from 220°C to 320°C. The Carr has shown an exothermic band at 220°C, which has been shifted to 230°C after treatment with Ch. This improvement could be attributed to the formation of a complex network between the Carr and the Ch. Similar behavior has been attained when the di-epoxy has been substituted with glutaraldehyde [3]. Further treatment with di-epoxy, Carr-Ch-Epo, gradually increased the temperature to two peaks at 250 and 230°C. This could be attributed to extra crosslinking with the di-epoxy and the two bands could be referred to the Ch and Epo; however, we could not tell at this stage which is which. However, by addition of Ch, Carr-Ch-Epo-Ch, the temperature increased to a single band at 260°C, which should be regarded to the Ch. That means that for the short chain, Carr-Ch-Epo, the two peaks should be for Ch and Epo, respectively. Finally, by adding more di-epoxy, Carr-Ch-Epo-Ch-Epo (long chain), two bands appeared for the Ch and Epo at the highest temperatures, 260 and 330°C, respectively.

On the other hand, the TGA of the modified Carr, formula numbers 2-5, showed a better stability against degradation as shown in Figure 3 and Table 1. For example, unmodified Carr had a sudden decomposition at 200°C. This value has been increased to 210°C after its treatment with Ch with retention of the sudden decomposition behavior. The gels' thermal improvement could be explained by the formation of polyelectrolyte interaction between the polyanions (–OSO_3_^−^) of the Carr and the polycations (NH_3_^+^) of the Ch. Further hardening of the gel beads using Epo showed a much higher increase in the TGAA Carr-Ch-Epo (short chain) to 250°C and the decomposition behavior was slower and gradual. The improvement in the TGA could be attributed to extra crosslinking between the free Ch's amino groups and the Epo. Further treatment of the short chain with Ch does not increase its TGA; however, it was more or less the same at 240°C. Finally, for the long chain, Carr-Ch-Epo-Ch-Epo, the TGA increased to its maximum of 270°C, which could be regarded to further crosslinking. It is worth noting that the short and long chains have the highest TGA values of 250 and 270°C with slower and gradual decomposition rate compared to other formulations.

### 3.2. Optimization of Enzyme's Loading Capacity

Two factors have been studied to optimize the loading capacity of *β*-galactosidases onto treated carrageenan gel beads.

#### 3.2.1. Effect of Epoxy Chain Length

**Figure 4:**
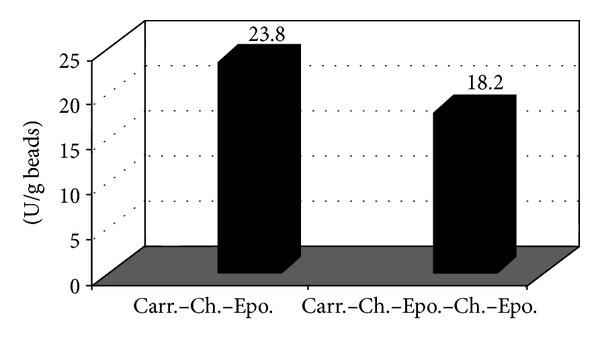
Effect of epoxy chain length on *β*-galactosidase loading capacity.

In this experiment short chain of epoxy modified gel beads, Carr-Ch-Epo, and long chain, Carr-Ch-Epo-Ch-Epo, were examined to assess chain length efficiency for immobilization of the enzyme. According to Sung and Bae, 2003 [22], the effect of the chain length could have a positive effect on the immobilization loading efficiency till certain length and then it declines afterwards. In our case, the short chain immobilized more enzymes than the long chain. Results as shown in Figure 4 showed that the short chain immobilized 23.8 U/g gel beds compared to 18.2 U/g gel beads for the long chain. These results are in accordance with that of Gancarz et al., 2003 [23], who observed that an increase in surface epoxy groups led to an increase in quantity of immobilized enzyme, but a decrease in retained enzyme activity.

To understand this phenomenon, we calculated from the supernatants the expected amounts of immobilized enzymes on the short and long chains formulas and they were found to be 18 and 21 U/g, respectively. These results were in favor of the long chain formula; however, the retained activity of the immobilized enzymes was in favor of the short chain, where 23.8 U/g were immobilized, showing 133% retention of activity. This increase in the enzyme activity after immobilization could be regarded to the hydrogen bond interactions between the modified gel (polysaccharide) containing –OH, –OSO_3_H, –C=O, –NH_2_, and the lactose substrate containing –OH, –C=O groups. These H-bonding interactions could also increase the lactose concentration surrounding the gel surface more than the bulk solution and thus the activity of the immobilized enzyme increases till reaching saturation of the gel surface with lactase [24].

On the other hand, the long chain formulation was expected to immobilize 21 U/g and in practice it showed only 18.2 U/g, which revealed 86% retention of activity of the immobilized enzyme. This could be regarded to the long chain having multipoint attachment or/and multilayers of the immobilized enzyme/steric hindrance that resulted in loss of the enzyme's 3D structure and consequently its activity. Accordingly, for further experiments, the short chain was used.

#### 3.2.2. Effect of *β*-Galactosidase Concentration

**Figure 5:**
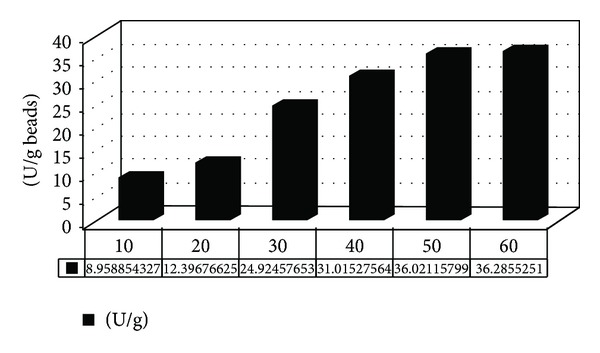
pH profile of the free and immobilized *β*-galactosidase.

*β*-Galactosidase was immobilized onto gel beads treated with short chain epoxy activated carrageenan, Carr-Ch-Epo, as shown in Figure 5.

Results revealed that by increasing the concentration of *β*-galactosidase from 10 U to 60 U, the E.L.C. increased gradually till it reached its maximum of 36 U/g gel beads using 50 U of free enzyme, after which any more added enzyme has almost no effect on the E.L.C. This could be regarded to all free epoxy groups that have been engaged with the enzymes [25]. However, we have chosen for further optimization the E.L.C. of 36 U/g gel beads as it shows better enzyme loading efficiency of 49%, which is more economic as it saves unloaded enzyme from being wasted.

### 3.3. Evaluation of Catalytic Activity of Free and Immobilized *β*-Galactosidase

At this stage, five experiments were studied. Firstly, the optimum reaction temperature, pH, and substrate concentrations were examined for both the free and immobilized enzyme. Secondly, the best results from the first step were used to obtain the maximum substrate hydrolysis as well as the operational stability of the immobilized enzyme.

#### 3.3.1. Optimum Temperature for the Free and Immobilized *β*-Galactosidase

**Figure 6:**
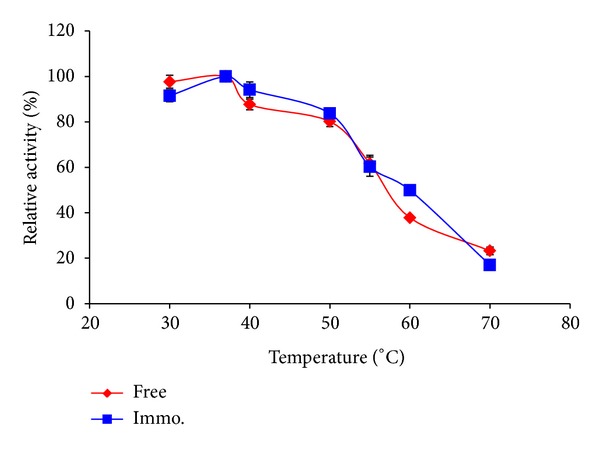
Michaelis constants of free and immobilized *β*-galactosidase.

The optimum temperature for the free and immobilized enzyme was examined. Results as shown in Figure 6 revealed that the optimum temperature for the immobilized enzyme was found to be at a slightly higher temperature (37–40°C) compared to the free enzyme (30–37°C).

The shift of the optimum temperature towards higher temperatures when the biocatalyst is immobilized indicates that the enzyme structure is strengthened by the immobilization process, and the formation of a molecular cage around the protein molecule (enzyme) was found to enhance the enzyme thermal stability. The increase of the immobilized enzyme temperature tolerance may also be due to diffusional effects where the reaction velocity is more likely to be diffusion limited, so that improvements in thermal diffusion would correspondingly result in proportionally higher reaction rates [3].

#### 3.3.2. pH Profile

**Figure 7:**
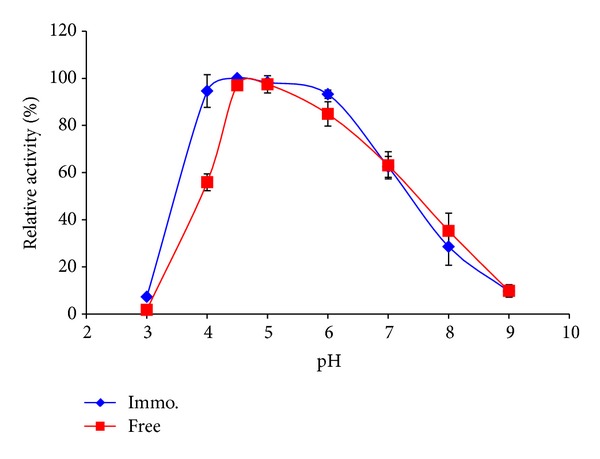
Lactose hydrolysis using free and immobilized *β*-galactosidase.

Figure 7 illustrates the pH activity profile of the free and immobilized *β*-galactosidase. The optimum pH values for free and immobilized enzyme were 4.5–5 and 4-6, respectively, which showed that the immobilized enzyme was more stable at higher and wider range of pH [25]. These properties could be very useful for lactolysis in sweet whey permeate, which has a pH range of 5.5–6. Moreover, at pH 4, the immobilized enzyme retained more than 95% of its relative activity compared to only 56% for the free enzyme.

The shift in the pH activity profile of the immobilized *β*-galactosidase and the better pH stability may be attributed to the partition effects that were arising from different concentrations of charged species in the microenvironment of the immobilized enzyme and in the domain of the bulk solution [3].

#### 3.3.3. Determination of Kinetic Parameters of Free and Immobilized *β*-Galactosidase

**Figure 8:**
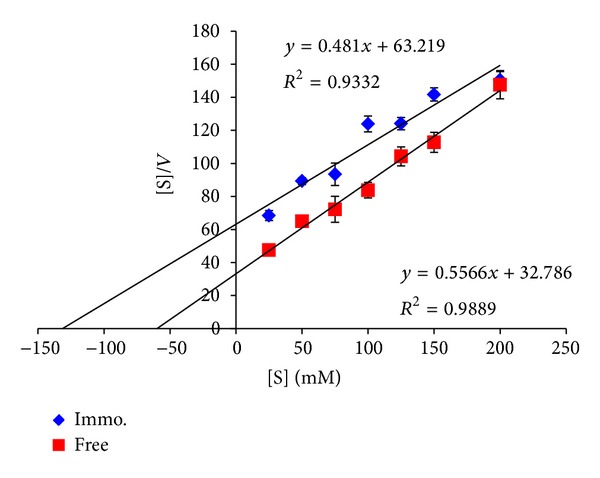
Reusability of the immobilized *β*-galactosidase.

The kinetic constants of free and immobilized *β*-galactosidase as shown in Figure 8 were tabulated in Table 2.

The apparent *K*_*m*_ after immobilization, 131.4 mM, is higher than that of the free enzyme, 58.9 mM, which indicates that a higher concentration of substrate, 2-fold, is needed for the immobilized enzyme compared to the free enzyme. Nevertheless, higher *K*_*m*_ values for immobilized *β*-galactosidase have been reported by other authors with increases from 1.2-fold up to 5.4-fold [26]. These results are most likely due to the fact that the immobilized enzyme surfaces are not accessible to all the reacting species. However, no substrate or product inhibition by the increase of substrate concentration up to 200 mM could be observed during our experiment, as shown by the straight line of the Hanes-Wolf representation (Figure 8).

On the other hand, the maximum reaction velocity, *V*_max⁡_, values for the immobilized enzyme were remarkable; it was found to double that of the free enzyme; that is, it increased from 32.7 to 63.2 *μ*mol·min^−1^. This result is in agreement with the speculation that the improvement in the immobilized enzyme thermal stability as in Section 3.3.1 could result in a higher reaction velocity. It is worth noting that the increase in the reaction velocity is generally favored in industries.

#### 3.3.4. Lactose Hydrolysis Using Free and Immobilized *β*-Galactosidase

This experiment has been carried out so that the immobilized enzyme could attain its maximum efficiency and act with its highest velocity using almost double the *K*_*m*_ concentration of substrate and using the enzyme's optimum conditions. A high concentration of substrate, 200 mM, at pH 4.5 and 37°C, was used in this study, as this enzyme was supposed to be suitable for hydrolysis of higher lactose concentrations found in mammal milk (88–234 mM lactose) and whey permeate (85% lactose) [25].

**Figure 9:**
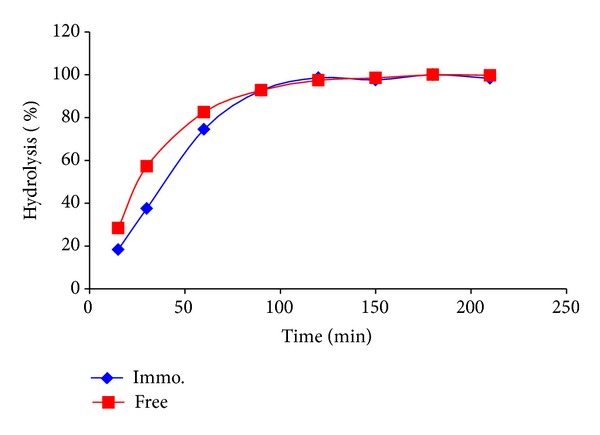
Effect of *β*-galactosidase concentration on the enzyme's loading capacity.

The results as shown in Figure 9 revealed that for the first hour, the rate of conversion of the free enzyme was higher than that of the immobilized one. This could be attributed to the fact that the gel needed longer time to reach its maximum swelling. This swelling will allow more substrates to penetrate into the pores and consequently decrease the diffusion limitation. However, at 90 min, both enzyme forms followed the same trend and the same speed till they reached maximum relative conversion at 120 min. It is worth noting that at 90 min, the gel beads carrying the enzyme reached its maximum swelling, overcame its substrate/product diffusion limitation, and followed the same trend as the free enzyme, which is advantageous in industries. That means that the small gel beads used in this work could overcome the problem the authors previously had when they used big gel disks as the enzyme suffered from diffusion limitation and hydrolyzed only 63% of the free enzyme [3].

#### 3.3.5. Reusability of Immobilized Enzyme

To evaluate the reusability of the immobilized enzyme, the beads were soaked in 200 mM lactose for 120 min till full conversion of lactose to glucose and galactose. The gel beads were removed from the product, washed with buffer solution after use, and then resuspended in a fresh aliquot of a substrate to measure the enzymatic activity.

**Figure 10:**
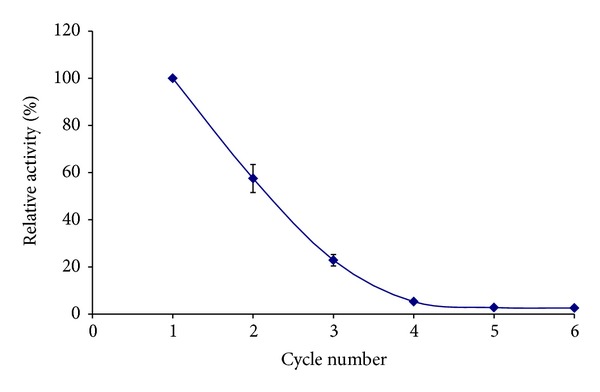
Temperature profile of free and immobilized enzyme.

This procedure was repeated until the enzyme lost its activity. The turn over number of the enzyme catalyzed process was calculated. As shown in Figure 10, the immobilized enzyme retained 60% of its relative activity by the second use and 21% by the 3rd use. Nevertheless, these results were in agreement with those obtained by other authors using the commercial carrier, Novozym 435, as the immobilized activity decreased to 23% after the second use and to 3.7% by the third use [27]. The loss in activity was attributed by other authors to inactivation of enzyme due to continuous use [28]. Although our carrier has shown better performance than that of Novozyme 435, we think that the modified gel with epoxy could be further modified for future work.

## 4. Conclusion

Novel biopolymer based on epoxy activated carrageenan was prepared for immobilization of lactase as an example of medical enzyme. The results were compared to those of our previous work, using aldehyde activated carrageenan. The epoxy formula showed far better immobilization efficiency that was triple that shown using the aldehyde one. That could be regarded to that the epoxy group is more active than the aldehyde group. The aldehyde group could only bind to the enzyme's free amino groups, whereas the epoxy group could bind to three groups, –SH, –NH_2_, and –OH. The results showed in Figure 9, hydrolysis of lactose, using free and immobilized lactase revealed that the immobilized enzyme could attain its maximum efficiency and act with its highest velocity as fast as the free enzyme. That was regarded to the gel beads carrying the enzyme that reached its maximum swelling and overcame its substrate/product diffusion limitation and followed the same trend as the free enzyme, which is advantageous in industries. The high activity of the epoxy formulation is highly recommended to be used for immobilization of other enzymes/proteins and/or drug delivery systems.

## Figures and Tables

**Table 1:** Values of DSC and TGAA thermograms of carrageenan and modified carrageenan with chitosan and epoxy groups.

Number	Formula	DSC temp, °C	TGA temp, °C
1	Carr	220	200
2	Carr-Ch	230	210
3	Carr-Ch-Epo	250 & 320	250
4	Carr-Ch-Epo-Ch	260	240
5	Carr-Ch-Epo-Ch-Epo	260 & 330	270

**Table 2:** Michaelis-Menten constants and maximal reaction rate values for free and immobilized lactase.

*β*-Galactosidase form	Kinetic constants	
	*K*_*m*_ (mM)	*V*_max⁡_ (*µ*mol·min^−1^)
Free	58.9	32.7
Immobilized	131.4	63.2

## Abbreviations

Carr: Carrageenan
Carr-Ch: Carrageenan-chitosan
Carr-Ch-Epo: Carrageenan-chitosan-epoxy
Carr-Ch-Epo-Ch: Carrageenan-chitosan-epoxy-chitosan
Carr-Ch-Epo-Ch-Epo: Carrageenan-chitosan-epoxy-chitosan-epoxy.
